# MicroRNA-34a Mediates the Autocrine Signaling of PAR_2_-Activating Proteinase and Its Role in Colonic Cancer Cell Proliferation

**DOI:** 10.1371/journal.pone.0072383

**Published:** 2013-08-26

**Authors:** Yiming Ma, Wuyun Bao-Han, Xue Lv, Yuntao Su, Xinhua Zhao, Yongmei Yin, Xingmao Zhang, Zhixiang Zhou, Wallace K. MacNaughton, Hongying Wang

**Affiliations:** 1 State Key Laboratory of Molecular Oncology, Cancer Institute/Cancer Hospital, Chinese Academy of Medical Sciences, Peking Union Medical College, Beijing, China; 2 Department of gastrointestinal cancer surgery, Cancer Institute/Cancer Hospital, Chinese Academy of Medical Sciences, Peking Union Medical College, Beijing, China; 3 First affiliated Hospital of Nanjing Medical University, Nanjing, PR China; 4 Inflammation Research Network, University of Calgary, Calgary, Alberta, Canada; 5 Department of Physiology and Biophysics, University of Calgary, Calgary, Alberta, Canada; Rush University Medical Center, United States of America

## Abstract

The tumor microenvironment is replete with proteinases. As a sensor of proteinases, proteinase activated receptor 2 (PAR_2_) plays critical roles in tumorigenesis. We showed that PAR_2_ and its activating proteinase were coexpressed in different colon cancer cell lines, including HT29. Inactivating proteinase or knockdown of PAR_2_ significantly not only reduced cell proliferation in vitro but also inhibited tumorigenicity of HT29 in vivo. In addition, activation of PAR_2_ promoted DNA synthesis and upregulated Cyclin D1 activity at both transcriptional and post-transcriptional levels. Further studies showed that miRNA-34a mediated PAR_2_-induced Cyclin D1 upregulation. Inhibition of miR-34a partially abolished the suppression of Cyclin D1 induced by PAR_2_ deficiency. In addition, we showed that TGF-β contributed to the regulation of miR-34a by PAR_2_. Finally, in colorectal carcinoma samples, upregulation of PAR_2_ and downregulation of miR-34a were significantly correlated with grade and lymphomatic metastasis. Our findings provide the first evidence that miRNA mediates autocrine proteinase signaling-mediated cancer cell proliferation.

## Introduction

With the completion of human genome project, a total of 553 genes are annotated to be encoding proteinase [1]. Identification of proteinase activated receptors (PARs) reveals a key role for proteinases, not only as protein-degrading enzymes, but also as potential receptor activators that transmit extracellular stimuli into intracellular signaling events. PARs are seven transmembrane-spanning domains G-protein coupled receptors (GPCRs) which act as targets of certain serine proteinases, such as thrombin and trypsin. Proteolytic cleavage of their extracellular amino terminus creates the new amino terminus which functions as a tethered ligand and activates the receptor. The unique activation mechanism of PARs provides a new mechanism by which microenvironment affects cell behavior. To date, four PAR family members have been identified, PAR_1_ to PAR_4_, all of which share similarities in their structure and activation mechanism [2]. PAR_2_ is the only member in the family which cannot be activated by thrombin. The studies in knockout mice revealed that PAR_2_ plays more important roles in tumor formation compared to other PARs [3].

PAR_2_ is widely expressed in the body and plays critical roles in various types of human cancer, including colon and lung cancer [4], [5]. PAR_2_ promotes tumor progression through a variety of mechanisms, such as cell proliferation, invasion and metastasis. It was shown that PAR_2_ stimulated cell proliferation in different cancer cells and emerged as a potent mitogenic factor in different cancers [6]–[10]. Further studies showed that transactivation of EGFR [7], activation of MAPK [9] and integrin α_5_β_1_-dependent adhesion to fibronectin [10] might mediate PAR_2_-stimulated cell proliferation. However, the molecular mechanisms by which PAR_2_ regulates the cell cycle are still obscure.

As an important component of the microenvironment, proteinases promote tumor cell proliferation and lead to uncontrolled cell growth. Some of the proteinases are able to act as endogenous activators of PAR_2_ in vivo. In addition to trypsin, urokinase-plasminogen activator (uPA)/plasmin, FXa, FVIIa, tissue factors, matriptase and kallikreins **(**KLKs) can all activate PAR_2_ [11]. Extra-pancreatic expression of trypsin is shown in gastric and colonic cancers [12], [13]. Forced expression of trypsinogen dramatically increases the tumorigenicity of gastric cancer cells in nude mice [13]. Direct evidence shows that trypsin acting on PAR_2_ is a very potent growth factor for human colon cancer cells [9].

Considering the omnipresence of PAR_2_, and the production of proteinases by tumors, the existence of an autocrine loop is not unexpected, especially in colorectal carcinomas. Abnormal expression of PAR_2_ was found in GI tract cancers and cancer cell lines [14]–[16]. Expression of matriptase and trypsin was detected in DLD-1 [17] and HT29 colonic cancer cell lines [18]. Most recently, KLK14 was found to be expressed and be able to activate PAR_2_ in colon cancer cells [19]. It is expected that the autocrine interaction of serine proteinases and PAR_2_ participates in cancer cell proliferate in the colon.

In the present study, we demonstrate that the autocrine action of trypsin and KLK14 promoted colon cancer cell proliferation through the activation of PAR_2_. Disruption of the autocrine loop by knockdown of PAR_2_ reduced cancer cell growth both in vitro and in vivo. Furthermore, we showed for the first time that miR-34a, which targets Cyclin D1, was essential for PAR_2_-induced cell proliferation.

## Methods and Materials

### Cell Culture and Cell Lines

The human colonic epithelial cell line HT-29, Caco-2, HCT-116, RKO and the human lung adenocarcinoma A549 cell line were obtained from ATCC (Manassas, VA, USA). The cells were grown in Dulbecco’s modified Eagle’s medium/F12 (Hyclone) supplemented with 10% FBS (Gibco).

### Stable Transfectant Cell Lines with PAR_2_ Knockdown

ShRNA vector pRFP-C-RS containing four different PAR_2_ specific interfering sequences and scrambled sequence were purchased from OriGene Technologies. HT29 cells were transfected with one of the shRNAs and selected with puromycin (1 µg/mL). Quantitative real-time PCR (qRT-PCR) and Western blot were performed to check the expression of PAR_2_.

### Chemicals and Reagents

The inhibitors for trypsin (Cat. No. T6522 and T9128) and actinomycin D were purchased from Sigma-Aldrich. Proteinase inhibitor cocktail (Cat. No. 04 693 159 001) and TGF-β were purchased from Roche and R&D respectively. E2F-driven luciferase reporter was generously provided by Professor Nathalie Rivard, University of Sherbrooke, Sherbrooke, QC, Canada. The PAR_2_-selective activating peptide (SLIGRL-NH_2_) and reverse-sequence inactive peptide (LRGILS-NH_2_) were prepared at Shanghai Apeptide Co. Ltd. (Shanghai, China). SiRNA for beta-catenin and control RNA were described previously [20].

### Ethics Statement and Human Samples

Tissue samples from thirty cases of colorectal cancer and matching normal mucosa were analyzed. The patients were recruited at the Cancer Hospital, Chinese Academy of Medical Sciences, Beijing, China. All patients received no anti-cancer treatment before surgery and signed informed consent forms for sample collection. All procedures involving human samples were approved by the Institutional Review Board of the Chinese Academy of Medical Sciences Cancer Institute. CRC tissues and paired normal tissues were submerged in RNAlater™ (Ambion) for 24 h and then stored at −70°C until use.

### BrdU Labeling Assay

Cell proliferation assay was done with BrdU labeling assay kit (Roche) according to the manufacturer’s procedure. Briefly, cells were incubated with 10 µM 5-bromo-2′-deoxyuridine (BrdU) for 24 h. After fixation, cells were incubated with anti-BrdU antibody for 1 h. Colorimetric analysis was done with an iMark™ microplate reader (BioRad) after the addition of substrate solution for about 20 min. The data were calculated according to the manufacturer’s instructions and shown as the absorbance value at 450 nm after the subtraction of the values for the blank group. The experiments for cell number were conducted parallel. The results of BrdU labeling assay were normalized with cell number.

### Colony Formation

For the colony formation assay, cells were seeded at low density (500 cells/plate) and allowed to grow till visible colonies appeared. The cells were then stained with Giemsa, and colonies were counted.

### MTT Analysis

3-(4,5-Dimethylthiazol-2-yl)-2,5-diphenyltetrazolium bromide (MTT) assays were conducted to measure the relative number of viable cells. At the indicated time points, medium was replaced with fresh medium supplemented with MTT (0.5 mg/ml) (Sigma-Aldrich). Absorbance was measured using iMark™ microplate reader (BioRad) at a wavelength of 490 nm. Experiments were repeated at least three times.

### Western Blot

Protein extracts from cultured cells were prepared by suspending cells in lysis buffer (0.01% EDTA, 0.1% Triton X-100, and 10% proteinase inhibitor cocktail). Protein concentrations were quantified using a protein assay kit (Bio-Rad). Briefly, 50 mg of lysates were separated on 12% SDS–PAGE and transferred to polyvinylidene difluoride membranes. The membranes were probed overnight at 4°C with primary antibodies against human Cyclin D1 (1∶1,000; Cell Signaling Technology), PAR_2_ (N-19, 1∶1000; Santa Cruz Biotechnology) or β-catenin (Cell Signaling Technology), followed by incubation with peroxidase-conjugated secondary antibodies (Cell Signaling Technology) at 1∶10,000 dilution for 1 h. The signal was visualized with ECL (Millipore).

### RNA Isolation, RT and PCR

Total RNA, isolated from A549 cells, HT29 cells or patient samples by using TRIzol reagent (Invitrogen), was treated with DNase I and reverse transcribed using a transcription kit (Invitrogen) to get total cDNA as templates for mRNA detection, or transcribed with TaKaRa™ microRNA transcription kit to get cDNA as templates for microRNA detection.

All the primers used for quantitative PCR were purchased from GeneCopoeia (Fulen Gen Co. Ltd., Guangzhou, China). Quantitative real-time PCR were normalized to GAPDH (glyceraldehyde-3-phosphate dehydrogenase) or U6 expression for mRNA and miRNA respectively.

Primers for regular PCR trypsinogen, KLK14 and PAR_2_ were as follows: trysinogen sense, 5′-TACCTTTGTTGCAGCTGCTG-3′, and anti-sense, 5′-CACCACCCACTGTTCGCTG-3′; KLK14 sense, 5′-CACTGCGGCCGCCCGATC; and anti-sense, 5′-GGCAGGGCGCAGCGCTCC-3′; PAR_2_ sense, 5′-TGAAGATTGCCTATCACATAC-3′ and anti-sense, 5′- TGCATTATTTTCTGATTAAGAGCC-3′. Actin was used as an internal control.

### Luciferase Reporter Assay

Transient transfection was performed with an E2F-driven luciferase as a reporter for transcriptional activity. The transfection agent Lipofectamine 2000 (Invitrogen) was incubated with DNA in serum-free media for 30 min before being added to cells. The cells were incubated with the transfection complex for 2 h. Cell lysates for luciferase activity were collected 24 h after transfection, and cells were treated with PAR_2_-AP for 3 h before harvesting. The luciferase assays were performed with a Dual-Luciferase assay kit (Promega) and data were normalized to pTK-RL.

### Tumorigenesis in Nude Mice

For in vivo studies, 10^6^ cells were injected into nude mice subcutaneously and tumor growth was followed for 3 weeks. All animal care was in accordance with the institutional guidelines. Tumor size (*V*) was calculated twice a week from the second week, using the formula, *V* (mm^3^) = 0.52×(width)^2^×(length).

### Statistical Analysis

Data are presented as means±SEM. Comparison of >2 groups was made using ANOVA with either a post hoc Tukey test or a post hoc Dunnett test. Comparison of 2 groups was made using Student’s t test for unpaired data. An associated value of *p<*0.05 was considered significant.

## Results

### Knockdown of PAR_2_ Blocked Cell Proliferation in HT29 Cells

**Figure 1:**
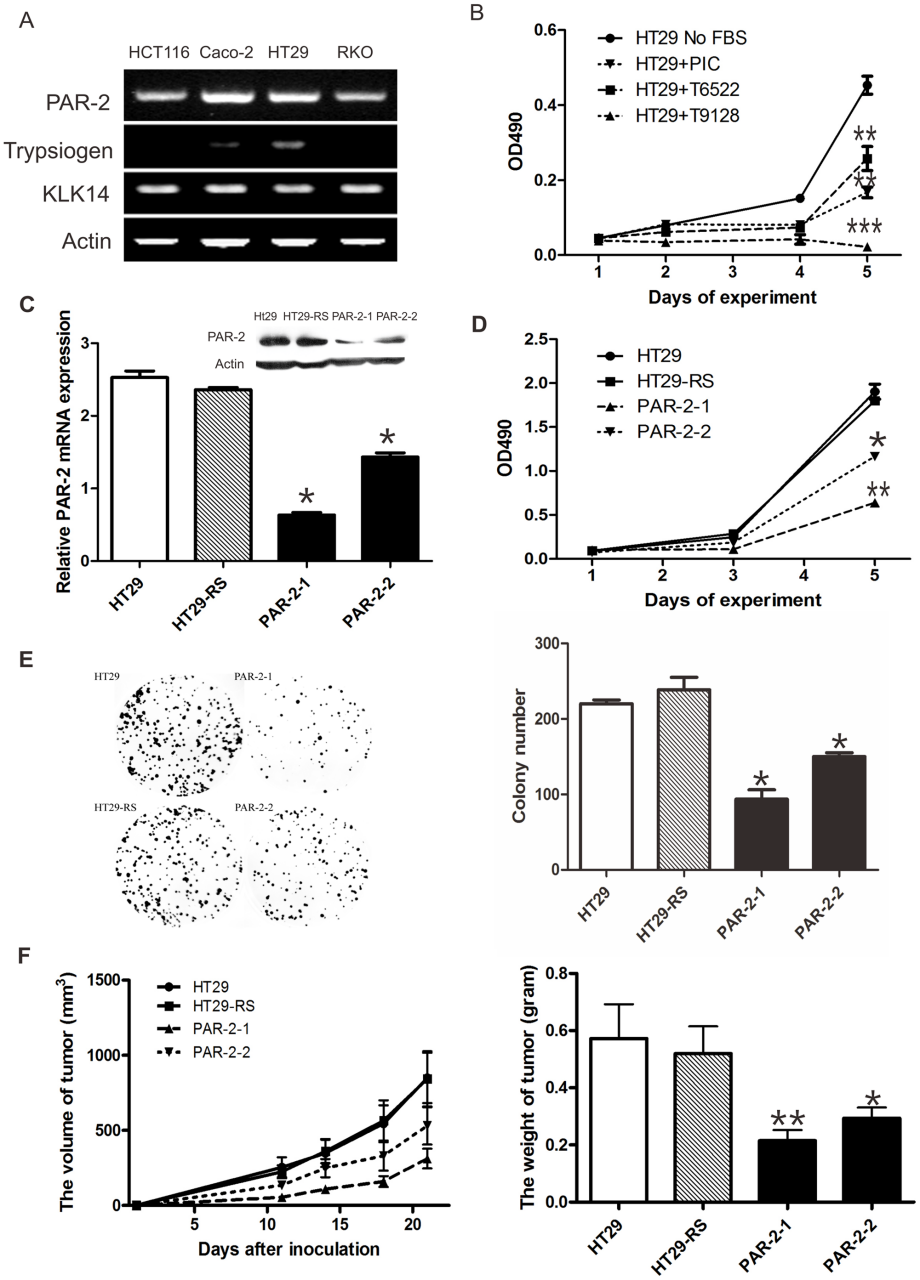
Knockdown of PAR_2_ blocked cell proliferation in HT29 cells. A. mRNA expression of PAR_2_, trypsinogen, KLK14 was measured with regular PCR in colon cancer cell lines. B. Colonic cancer cells were pretreated with the inhibitors of trypsin (T9128 and T6522) or serine proteinases (proteinase inhibitor cocktail, PIC) and cell proliferation was measured by MTT assay. C. The mRNA expression of PAR_2_ in stable transfectant cells (PAR_2_-1 and PAR_2_-2) was measured by real time PCR. HT29 RS represents the control cells stably transfected with scrambled control RNA. Protein level of PAR_2_ was measured by Western Blot and shown as insert. The effect of PAR_2_ knockdown on cell proliferation was measured by (D) MTT assay and (E) colony formation in HT29 cells. F. Cells (10^6^) were injected subcutaneously into nude mice, tumor volumes were measured as indicated and tumor weights were determined at sacrifice. *p<0.05, **p<0.01 All data are shown as mean±SEM. N = 3–6. All experiments have been repeated at least 3 times independently.

In this study, we tested PAR_2_ mRNA levels in different colon cancer cells including HT29, HCT116, Caco-2 and RKO cells. Consistent with previous reports [18], all the cells tested showed positive expression of PAR_2_ (Figure 1A). The expression of endogenous PAR_2_-activating proteinases trypsinogen and KLK14 were also investigated by RT-PCR. KLK14 mRNA was present in all human colon cancer cells analyzed, while trypsinogen expression was observed only in Caco-2 and HT29 cell lines (Figure 1A). Since HT29 cells present high expression of both trypsinogen and KLK14, we selected this colon carcinoma-derived cell line for further study.

To determine whether the PAR_2_-proteinase autocrine loop is crucial for cell proliferation, we used two methods to break down the autocrine loop. First, different inhibitors were used to block the activity of proteinase. Two trypsin inhibitors (T6522 and T9128) and a proteinase inhibitor cocktail (PIC) were applied to block known and unknown serine proteinase which can activate PAR_2_, and all of them significantly blocked HT29 cell proliferation as measured by the MTT assay (Figure 1B). Second, PAR_2_ expression was stably knocked down in HT29 cells (Figure 1C). MTT and colony formation analysis indicated respectively that knockdown of PAR_2_ blocked cell proliferation (Figure 1D) and colony-forming ability (Figure 1E) in vitro. Most importantly, knockdown of PAR_2_ in HT29 cells significantly reduced tumor growth in nude mice (Figure 1F). These data indicated that autocrine activation of PAR_2_ by its activating proteinase promote colon cancer cell proliferation both in vitro and in vivo.

### Cyclin D1-E2F Pathway Mediated PAR_2_-mediated Cell Proliferation

**Figure 2:**
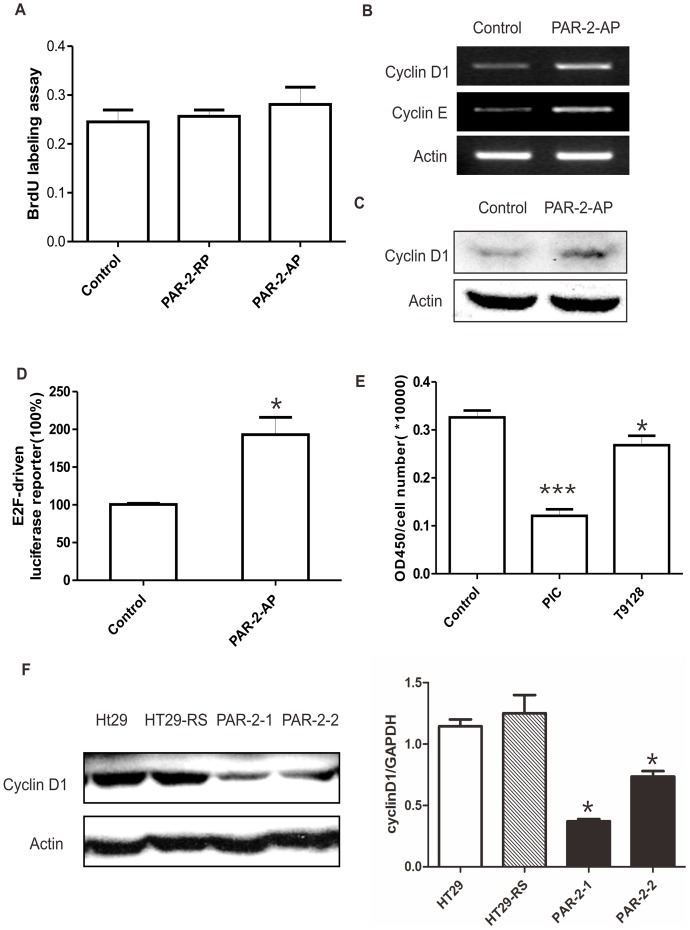
Activation of PAR_2_ increases cell proliferation through Cyclin D1-E2F pathway. A549 cells were treated with PAR_2_ activating peptide (PAR_2_-AP, 50 µM) or reverse peptide (PAR_2_-RP) for 24 h. A. DNA synthesis was measured by BrdU labeling assay.B. Levels of Cyclin D1 and Cyclin E mRNA were detected with PCR after treatment with or without PAR_2_-AP. C. Protein level of Cyclin D1 was determined by Western blot. D. A549 cells were treated with PAR_2_-AP (50 µM) 24 h after transient transfection with E2F-luciferase (1 µg/well). The data were normalized with tk-RL and shown as the percentage of the control group. E. HT29 cells were treated with PIC (proteinase inhibitor cocktail) or T9128 (trypsin inhibitor). DNA synthesis was measured by BrdU labeling assay. F. Protein (left) and mRNA (right) levels of Cyclin D1 in PAR_2_ knockout cells were measured. *p<0.05, ***p<0.001 All data are shown as mean±SEM. N = 3–6. All experiments have been repeated at least 3 times independently.

To determine the molecular mechanisms underlying the effect of PAR_2_ activation on proliferation, we used the human lung carcinoma-derived A549 epithelial cell line, which expresses PAR_2_ but not the activating proteinase. The BrdU labeling assay showed that the selective PAR_2_-activating peptide (PAR_2_-AP), but not the reverse-sequence peptide (RP), induced a significant increase in DNA synthesis (Figure 2A). Furthermore, PAR_2_ activation significantly increased Cyclin D1 at both the mRNA and protein levels (Figure 2B and C). These results suggest that PAR_2_ activation promotes cell cycle at the G1/S checkpoint.

In general, activation of the Cyclin/CDK complex can cause hyperphosphorylation of pRb which leads to E2F transcriptional activation and turn on the downstream target genes, such as Cyclin E. The luciferase reporter assay showed that PAR_2_-AP activation of PAR_2_ elevated E2F transcriptional activity (Figure 2D), along with a concurrent elevation of Cyclin E mRNA expression (Figure 2B). These results indicate that PAR_2_ activation promoted cell proliferation through Cyclin D1/E2F signaling.

Furthermore, we tested whether Cyclin D1 plays a role in PAR_2_ auto-activation in HT29 cells. Treatment with PIC or trypsin inhibitor significantly abolished DNA synthesis in HT29 cells (Figure 2E). The expression level of Cyclin D1 in PAR_2_ knockdown cells was also downregulated at both the protein and mRNA levels (Figure 2F). These findings suggest that Cyclin D1 mediated PAR_2_-induced cell proliferation.

### PAR_2_ Regulates Cyclin D1 Expression at Transcriptional and Post-transcriptional Levels

**Figure 3:**
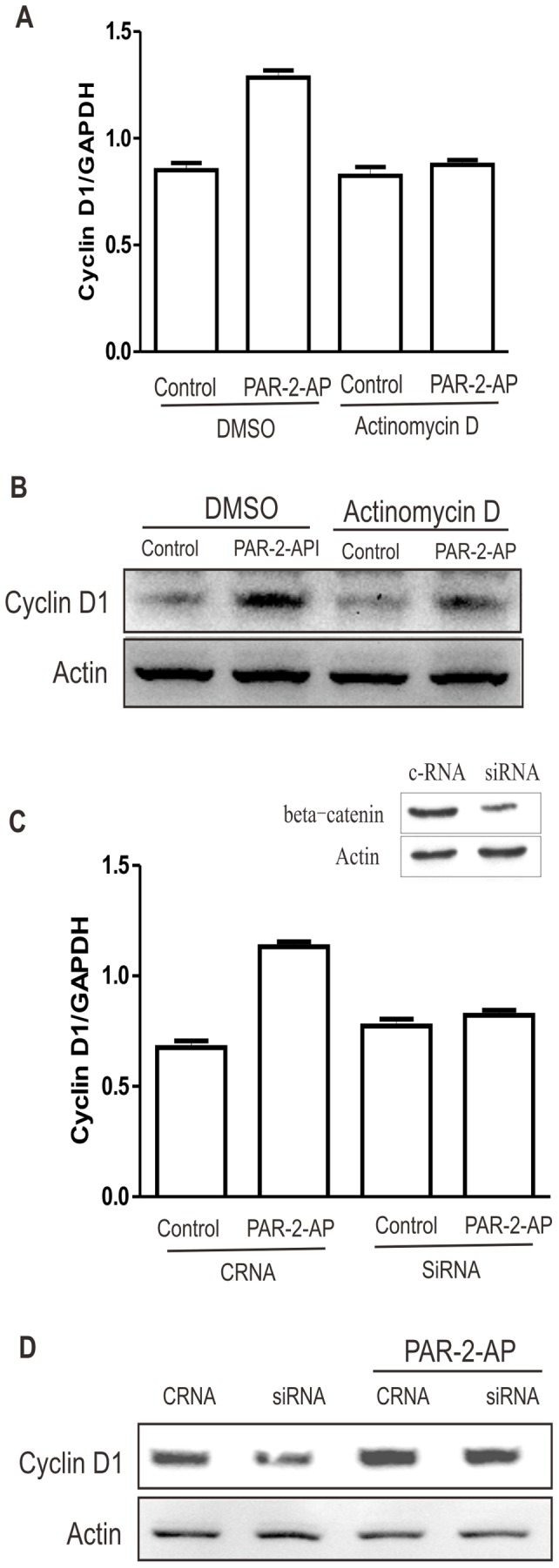
PAR_2_ regulates Cyclin D1 expression at transcriptional and post-transcriptional levels. A549 cells were pretreated with actinomycin D (2 µg/ml) 30 min before challenge with PAR_2_-AP (50 µM) for 24 h. (A) mRNA and (B) protein levels of Cyclin D1 were detected by real time PCR and Western Blot. siRNA targeting beta-catenin was transfected into A549 cells 24 h before treatment with PAR_2_-AP. (C) mRNA and (D) protein levels of Cyclin D1 were detected by real time PCR and Western Blot, respectively.

To understand the mechanism by which PAR_2_ activation regulates Cyclin D1 expression, we used A549 cells which have no autocrine loop. First, A549 cells were pretreated with actinomycin D to inhibit the transcription. Inhibition of transcription completely blocked PAR_2_-induced Cyclin D1 at the mRNA level (Figure 3A), but not at the protein level (Figure 3B). On the other hand, knockdown of β-catenin, which is a key transcription factor for the induction of Cyclin D1, also completely blocked Cyclin D1 mRNA expression (Figure 3C). These findings suggested that β-catenin mediated the PAR_2_-induced upregulation of Cyclin D1 at the transcriptional level. However, knockdown of β-catenin only modestly reduced Cyclin D1 at the protein level (Figure 3D), indicating that Cyclin D1 was also upregulated by PAR_2_ at post-transcriptional level.

It has been reported that phosphorylation of Cyclin D1 is involved in its degradation. However, there was no significant change in phospho-Cyclin D1 after PAR_2_ activation (data not shown).

### MiRNAs Mediate PAR_2_-induced Cyclin D1 Expression

**Figure 4:**
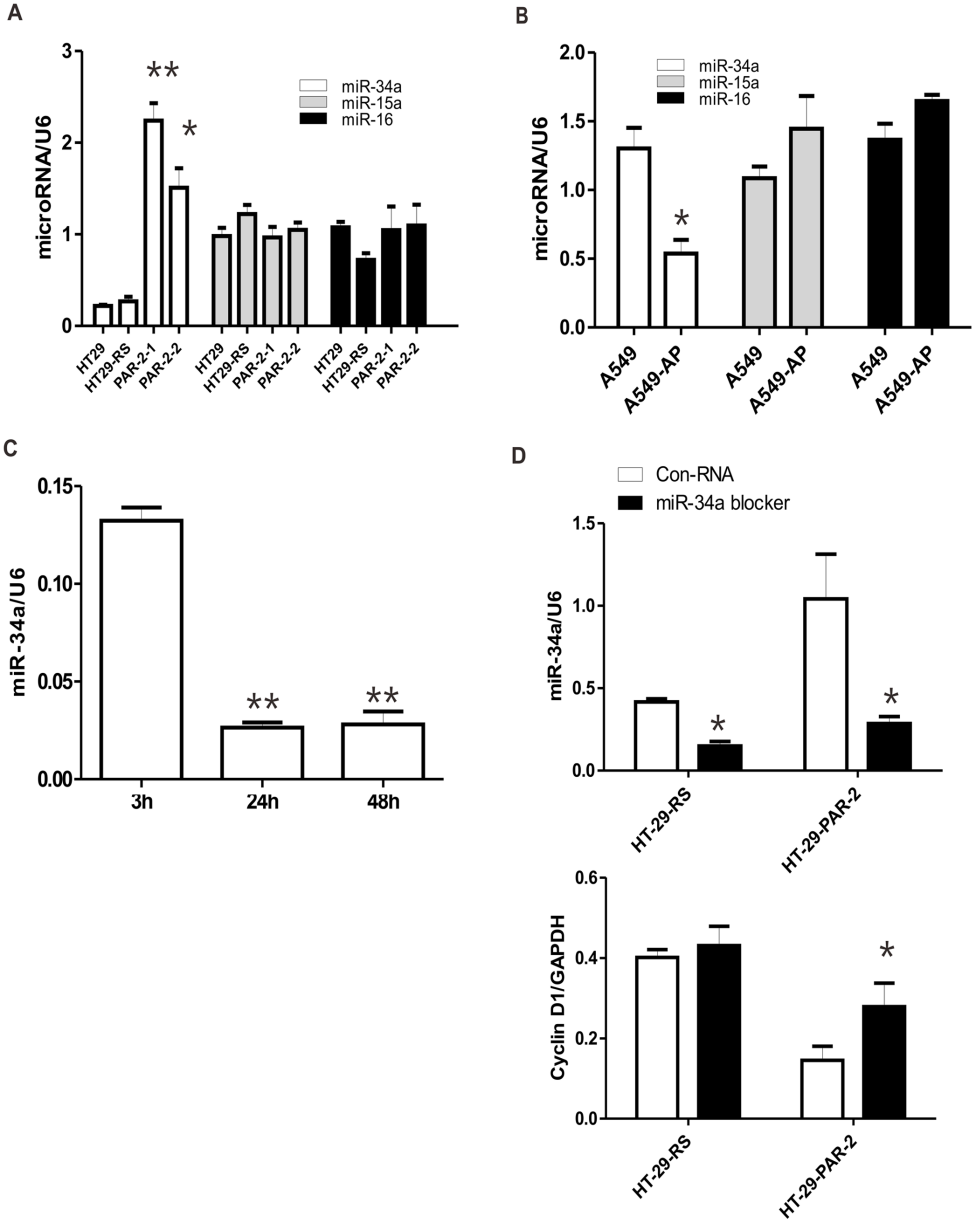
MiRNAs involved in PAR_2_-related Cyclin D1 expression. (A) Total RNA of HT29, HT29 RS (scrambled control RNA) and two different clones of stable transfectant cells with PAR_2_ knockdown (PAR_2_-1 and PAR_2_-2) were collected. The levels of targeted miRNAs were measured with real time PCR. (B) A549 cells were treated with PAR_2_-AP for 24 h. (C) HT29 cells were incubated with DMEM/F-12 medium without FBS for different time as indicated. (D) Transfection of miR-34a inhibitor or control RNA(c-RNA) with Lipofectamin2000 into HT-29-RS or HT-29-PAR-2. Three days later the cells were collected for assay. The levels of miRNA were measured with real time PCR and normalized with U6. *p<0.05, **p<0.01 All data are shown as mean±SEM. N = 3–6.

In addition to protein modification, microRNAs (miRNAs) are emerging as important regulators of gene expression by cleaving target mRNA or by inhibiting their translation. Indeed, several microRNAs had been demonstrated to regulate Cyclin D1 expression directly [21]. To determine whether miRNAs were regulated by PAR_2_, we detected the levels of miR-15a, miR-16, and miR-34a in PAR_2_ knockdown cells and PAR_2_ activated cells. Real time PCR showed that the level of miR-34a, but not mir-15a and miR-16, was dramatically elevated in PAR_2_ knockdown HT29 cells (PAR_2_-1 and PAR_2_-2) compared with scrambled RNA control cells (RS) (Figure 4A). Furthermore, PAR_2_ activation significantly decreased the miR-34a expression in A549 cells (Figure 4B). To test whether miR-34a can be regulated by HT29-derived proteinases, we starve HT29 cells for 3 h, 24 h and 48 h to accumulate the PAR_2_-activating proteinases (trypsin and KLK14) in medium. Quantitative PCR showed that miR-34a expression level was downregulated with time (Figure 4C). Inhibitor of miR-34a which reduced the level of miR-34a (Figure 4D, upper panel) partially restored the downregulation of Cyclin D1 in PAR_2_ knockdown cells (Figure 4D, lower panel). Taken together, these data suggest that miR-34a mediated PAR_2_-induced upregulation of Cyclin D1.

### TGF-β Mediated PAR_2_-related miRNA-34a Expression

**Figure 5:**
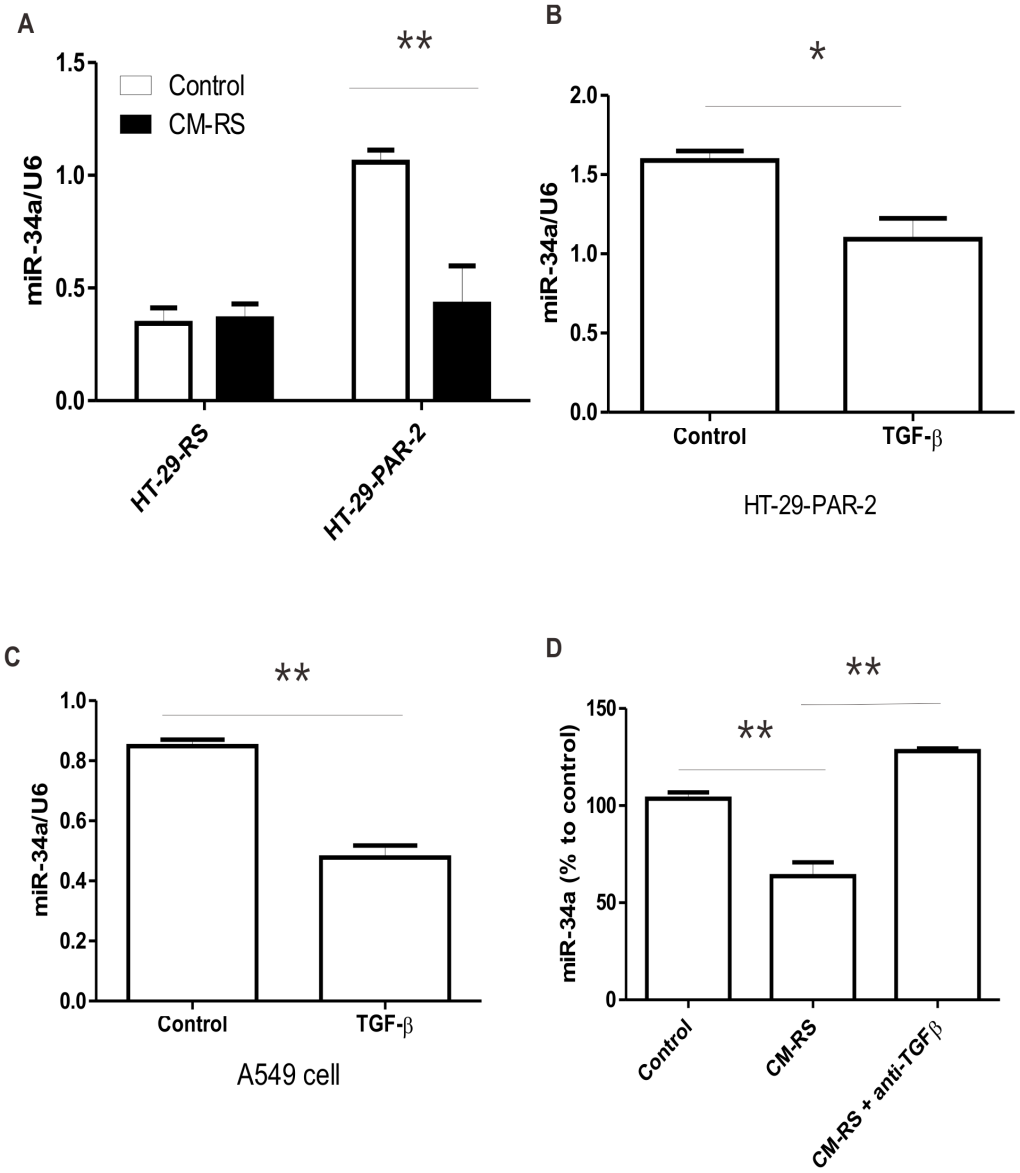
TGF-β mediated PAR_2_-related miRNA-34a expression. (A) HT-29-RS or HT-29-PAR-2 cells were treated with conditional medium from HT-29-RS cells (CM-RS) for 3 days. (B) HT-29-PAR-2 cells were treated with TGF-β (5 ng/ml) for 12 hours. (C) A549 cells were treated with TGF-β (5 ng/ml) for 6 hours. (D) Conditional medium from HT-29-RS (CM-RS) was incubated with anti-TGF β antibody and protein A/G agarose at 4°C for 2 hours. After centrifuge, the supernatant (CM-RS-anti-TGF β) was used to treat HT-29-PAR-2 cell for 3 days. After the different treatments as mentioned above, the cells were collected for the assay of miR-34a with real time PCR. *p<0.05, **p<0.01 All data are shown as mean±SEM. N = 4–8.

The next question is how PAR_2_ activation regulates the expression of miR-34a. The treatment with conditional medium from control HT29 cells (HT-29-RS) significantly reduced the expression level of miR-34a which was upregulated by knockdown of PAR_2_ in HT29 cells (HT-29-PAR-2) (Figure 5A). Since TGF-β has been reported to decrease the expression of miR-34a recently (39), the role of TGF-β in PAR-2-related miR34a expression was tested. Firstly, we found that TGF-β (5 ng/ml) was able to decrease miR-34a expression both in PAR_2_-deficient HT29 cells (HT-29-PAR2) (Figure 5B) and in A549 cells (Figure 5C). Most importantly, depletion of TGF-β from the conditional medium of HT-29-RS by anti-TGFβ antibody abolished inhibitory effect of conditional medium on miR-34a expression (Figure 5D). All these strongly indicated that TGF-β mediated the regulation of miR-34a induced by PAR_2_ activation.

### Correlation of PAR_2_, Trypsinogen, KLK14, Cyclin D1 and miR-34a in Colon Cancer Samples

**Figure 6:**
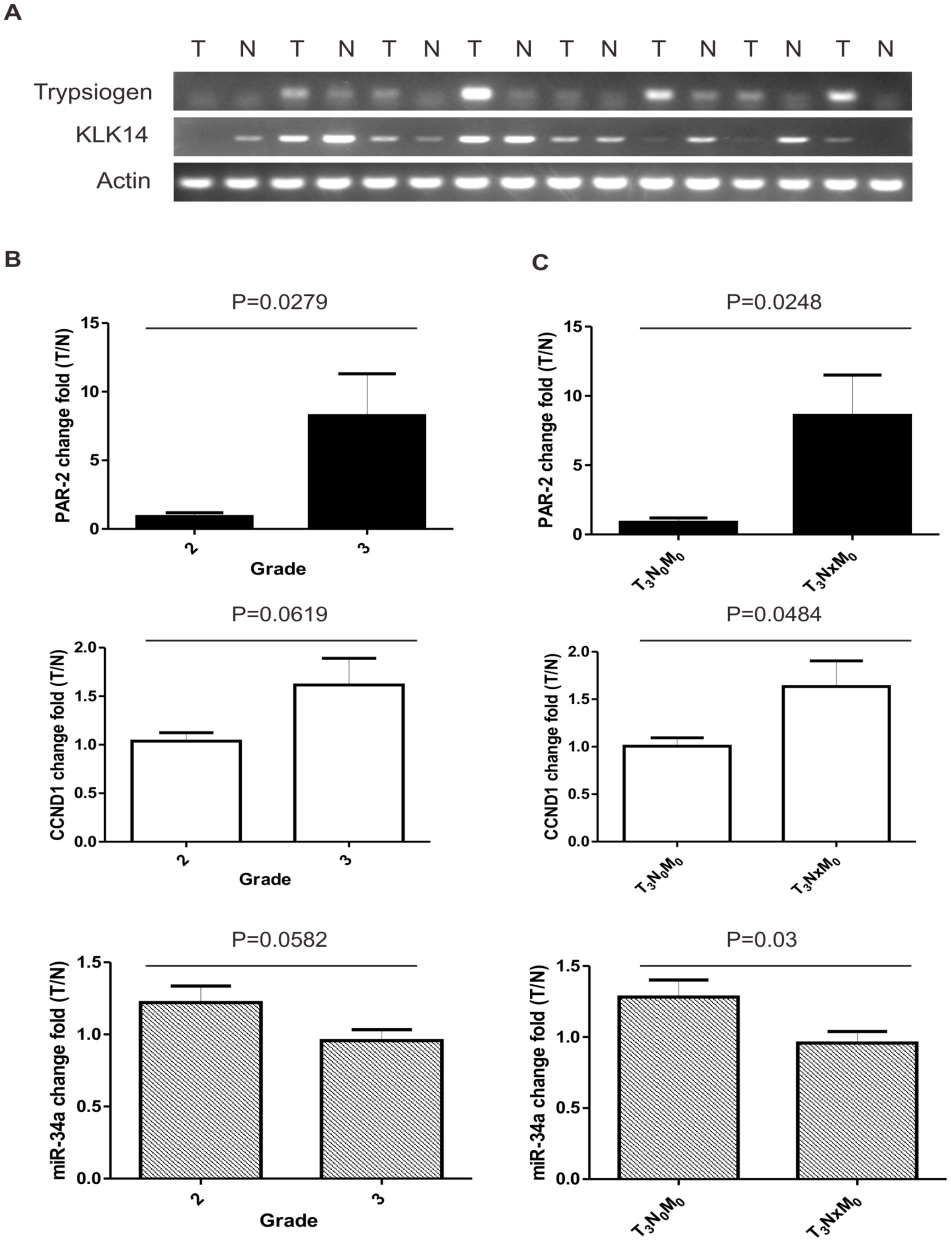
Expression of PAR_2_, CCND1 and miR-34a in human samples. (A) The expression of trypsinogen and KLK14 in human CRC tumor samples (T) and paired normal tissue (N) were measured with PCR. Actin was used as an internal control. The mRNA expression of PAR_2_, CCND1 and the level of miR-34a in human CRC samples were tested with real time PCR. They were compared according to (B) the grade or (C) the status of lymph node metastasis. The data are shown as the fold change of tumor sample (T) to paired normal tissue (N).

To gain insight into the biological role of PAR_2_ autocrine signaling in human colorectal carcinogenesis, expression levels of PAR_2_ and its activating proteinase were measured in 30 paired human T3 colorectal cancer (CRC) samples. Regular RT-PCR was employed to detect mRNA expression of trypsinogen and KLK14 in CRC samples and showed that almost all the samples express PAR_2_-activating proteinase (Figure 6A). Seventy percent (21/30) of human CRC samples showed elevated trypsinogen expression in tumor tissues compared with paired normal colon tissues, while normal tissues showed almost no trypsinogen expression. Real time PCR demonstrated that PAR_2_ was widely expressed in normal mucosa and primary CRC samples. As depicted in Figure 6B, PAR_2_ was found to be, on average, significantly (*p*<0.05) overexpressed in CRC samples with higher development grade, whereas CyclinD1 and miR-34a showed no significant changes (Figure 6B). Interestingly, higher levels of PAR_2_, Cyclin D1 and lower level of miR-34a were significantly correlated with lymph node metastasis in CRC samples (Figure 6C). Taken together, these data validated that the autocrine loop of PAR_2_ and its activating proteinase may promote tumor development and cancer invasion in human CRC samples.

## Discussion

MicroRNAs (miRNAs) are endogenously expressed 17–25 nucleotide, non-coding RNAs and regulate gene expression at post-transcriptional level. Since the first miRNA, lin-4, was discovered in 1993 [22], [23], approximately 1000 miRNAs have been identified in humans [24] and may target over 30% of all genes and a majority of genetic pathways [25], [26]. In the last decade, miRNAs have emerged as critical regulators in cancer [27] and are involved in the development of a range of cancers, including colorectal carcinoma [28]. Increasing evidence indicates that specific miRNAs are correlated with disease stage, metastasis and survival in CRC. To date, there are 118 miRNAs all of which have been verified and are associated with CRC development [29]. Some of miRNAs function as tumor suppressor, such as let7a, miR-34 a/b/c, miR-126, miR-143, miR-145, and miR-200 family, while some of them are thought to be oncogenic, such as the miR-17-92 cluster, miR-21, and miR-135.

**Figure 7:**
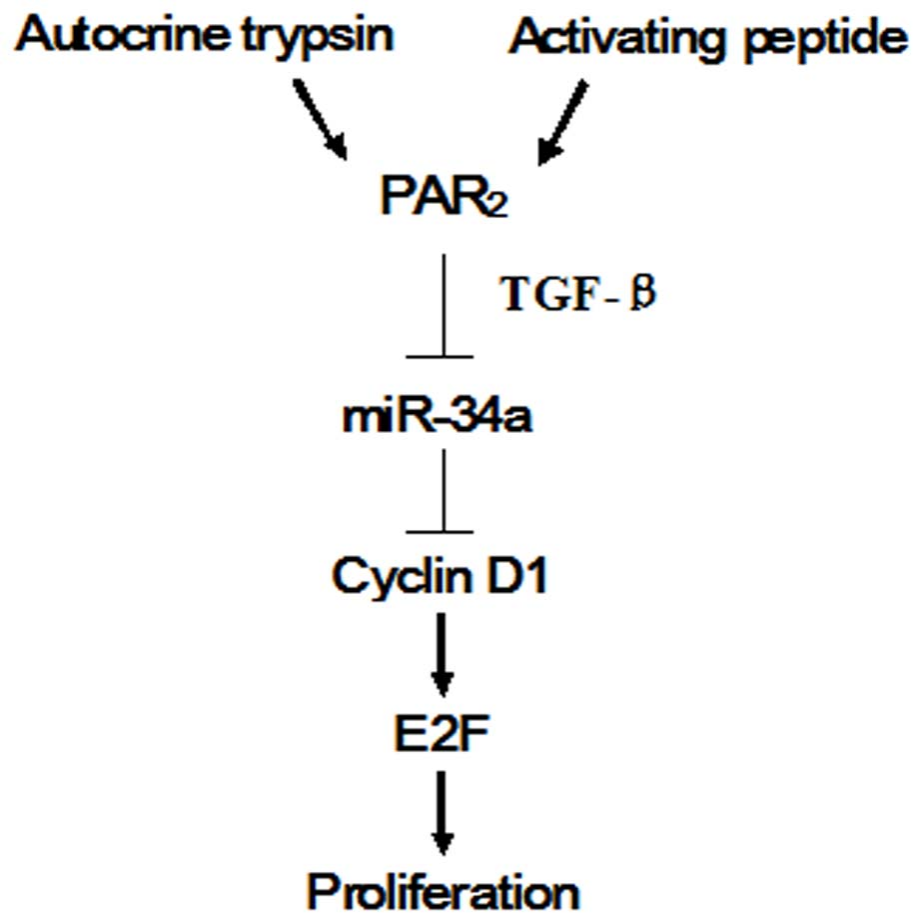
The overall pathway by which the activation of PAR_2_ regulates cell proliferation.

Uncontrolled cell proliferation is a feature of cancer. A number of miRNAs have been identified that regulate cell cycle. Some of the miRNAs promote cell proliferation, such as miR-125b [30], while some of them induce cell cycle arrest. For example, Let-7 has been found to be a potent growth suppressor in different cancer cells [31], [32]. Two related miRNAs miR-143 and miR-145 have been found to suppress growth partly through targeting ERK5 [33]. Thrombin has been shown to stimulate cell proliferation through the increase of miR-222 which can inhibit p27 expression [34]. The current study demonstrated that serine proteinase activated PAR_2_ and was also able to affect the level of miRNAs, which functionally mediated cell growth in cancer cells (Figure 7).

As a key regulator of G1/S checkpoint, Cyclin D1 can be targeted by various miRNAs. MiR-15a and miR-16 are frequently deleted or down-regulated and these events inversely correlate with the expression of Cyclin D1 in cancer. Further studies showed that Cyclin D1 is directly regulated by miR-15a and miR-16 [35]. In addition, Cyclin D1 expression can be regulated by miR-200b and Let-7b by targeting Rho family GTPase 3 (RND3) [36], [37]. In the current study, miR-15a and miR-16 were detected. However, there was no significant change in their expression levels after PAR_2_ activation in A549 cells (Figure 3F) or after PAR_2_ knowdown in HT29 cells. Moreover, there was no significant change of miR-200b and Let-7b (data not shown), nor was there any consistent change in the expression of the target gene, RND3, as determined by western blot and luciferase reporter assay with 3′ UTR of RND3 (data not shown). It is implied that miR-15a, miR-16 and miR-200b are not the mediators of PAR_2_-induced upregulation of Cyclin D1.

MiR-34a belongs to miR-34 family which also includes miR-34b and miR-34c. Low levels of miR-34 have frequently been observed in various tumors, including CRC [38]. Inactivation of endogenous miR-34a stimulates cell proliferation and inhibits p53-dependent apoptosis through targeting Cyclin E2, Cyclin-dependent kinases 4/6 (CDK4/6) [38]. Our study has shown, for the first time, that miR-34a mediated the autocrine loop of PAR_2_ and its activating proteinase, which is required to maintain colonic cancer cell proliferation.

Activation of ERK and transactivation of EGFR have been shown to be involved in the proliferative effect of PAR_2_ [7], [9]. However, in the current study, neither the inhibitor of ERK (PD98059 and U0126) nor the inhibitor of EGFR tyrosine kinase (AG1478) were able to block the cell proliferation or the change in miR-34a expression in HT29 and A549 cells (data not shown). It implied that the regulation of miR-34a was not dependent on EGFR and ERK pathway after PAR_2_ activation in cancer cells.

Recently, it has been reported that elevated TGF-β activity suppressed the expression of microRNA-34a in the liver tissue [39]. In addition, PAR_2_ activation has been demonstrated to augment TGFβ production in mice [40]. Therefore, it is not surprised that TGFβ mediates the suppression of miR-34a induced by PAR_2_ activation in current study. Furthermore, constitutive activation of PAR_2_ due to the autocrine loop of PAR_2_ and its activating proteinase may partially contribute to the low level of miR-34a, which is common in various cancers, such as colon cancer.

Given the emerging roles of PAR_2_ in cancer, PAR_2_ has become attractive target for the cancer therapy. PAR_2_ was broadly expressed in cancer and positively correlated with tumor progression in CRC. More importantly, PAR_2_ was activated not only by proteinases from the microenvironment of cancer, but also, as our study shows, by proteinases produced by cancer cells themselves. Moreover, considering the regulation of miRNA by PAR_2_ activation that we have now described, a more extensive effect on proliferation will be expected after targeting PAR_2_ activation in cancer cells. With the improvement of PAR_2_ antagonists currently under development, PAR_2_ will be a good target for cancer therapy in the future.
